# Mental health care use and related factors in adolescents and young adults with cancer

**DOI:** 10.1007/s00520-023-07708-4

**Published:** 2023-03-31

**Authors:** Takatoshi Hirayama, Satoru Ikezawa, Ryo Okubo, Tomoko Mizuta, Shintaro Iwata, Tatsuya Suzuki

**Affiliations:** 1 Department of Psycho-Oncology, National Cancer Center Hospital, 5-1-1 Tsukiji, Chuo-ku, Tokyo, Japan; 2 Department of Psychiatry, National Center of Neurology and Psychiatry Hospital, Tokyo, Japan; 3 Endowed Institute for Empowering Gifted Minds, Graduate School of Arts and Sciences, The University of Tokyo, Tokyo, Japan; 4 Department of psychiatry and neurology, National hospital organization Obihiro hospital, Hokkaido, Japan; 5 Department of Musculoskeletal Oncology and Rehabilitation, National Cancer Center Hospital, Tokyo, Japan; 6 Department of Hematology, National Cancer Center Hospital, Tokyo, Japan

**Keywords:** Adolescent and young adult, Cancer, Mental health, Psychological support

## Abstract

**Purpose:**

The actual state of mental health care use and related factors in adolescent and young adult (AYA) patients with cancer is not well understood in Japan. This study aimed to (1) examine the actual state of mental health care use among AYA patients with cancer and (2) describe socio-demographic and related factors associated with mental health care use.

**Methods:**

We retrospectively reviewed the medical records of AYA patients with cancer aged 15–39 who first visited the National Cancer Center Hospital in Japan (NCCH) between January 2018 and December 2020. Logistic regression was used to analyze the association between social background characteristics and mental health care use. The association between the patient's course of cancer treatment and mental health care use was analyzed to help identify which patients might benefit from early mental health intervention.

**Results:**

Among 1,556 patients, 945 AYA patients with cancer were registered. The median age at the time of the study was 33 years (range, 15–39 years). The prevalence of mental health care use was 18.0% (170/945). Age 15–19 years, female gender, urogenital cancer, gynecological cancer, bone or soft tissue cancer, head and neck cancer, and stage II–IV disease were associated with mental health care use. Regarding treatment, palliative treatment, chemotherapy, and hematopoietic stem cell transplantation were associated with mental health care use.

**Conclusion:**

Factors associated with mental health care use were identified. Our findings potentially contribute to psychological support interventions for AYA patients with cancer.

## Introduction

Adolescent and young adult (AYA) patients with cancer are defined as those aged 15 to 39 years at the time of initial cancer diagnosis [1]. In the United States, approximately 87,000 AYAs are newly diagnosed with cancer each year, approximately 4.5% of all people diagnosed with cancer [2]. In Japan, approximately 20,000 AYAs are newly diagnosed with cancer each year, approximately 2.3% of all people diagnosed with cancer [3].

AYA patients with cancer are complex and vulnerable as a result of the intersection of disease and developmental stage [4]. AYA patients with cancer are in various challenging situations related to physical and cognitive development, identity, body image, autonomy, and employment [5, 6]. A diagnosis of cancer could significantly disrupt or delay these aspects of development.

Cancer treatment can interfere with education or employment plans [7], which might cause great distress for AYA patients with cancer. Research indicates that AYA patients with cancer report significantly poorer physical and mental health than matched controls [8] and that young adult patients with cancer report more negative psychosocial outcomes than older patients [9].

Previous questionnaire surveys have evaluated psychological distress and related factors in AYA patients with cancer. The related factors were reported to be younger or older age, female gender, digestive system cancer, breast cancer, head and neck cancer, chemotherapy or radiotherapy, not being in a partnership, being divorced, lower monthly income, lower education level, lower exercise intensity, longer time since diagnosis, anxiety, depression, and cancer- and treatment-related adverse late outcomes [10–13].

In a questionnaire survey in Japan, pain, decrease in income after a cancer diagnosis, experience of negative changes at work or school after a cancer diagnosis, and poor social support were reported to be significantly associated with psychological distress [14]. Furthermore, AYA patients with cancer in Japan have a three-fold higher risk of major depressive disorder within 6 months before and 12 months after cancer diagnosis compared with cancer-free controls [15]. A previous study found that AYA patients with cancer aged 15–24 years have a higher risk of suicide than cancer-free controls [16].

The international clinical practice guidelines from the International Late Effects of Childhood Cancer Guideline Harmonization Group [17] strongly recommend mental health surveillance for all survivors of childhood, adolescent, and young adult cancers at every follow-up visit and prompt referral to mental health specialists when problems are identified. The recommendations reflect the necessity of mental health surveillance as part of comprehensive survivor-focused health care.

In a previous study [6], AYA patients with cancer were more likely to demonstrate psychological distress than individuals without cancer. Nevertheless, few survivors might be receiving professional mental health care. Survivors need great access to mental health screening and counseling to address the current gaps in care delivery. A previous study reported that there is a strong need for psycho-oncological interventions designed to improve mental health in AYA patients with cancer at all stages of medical care [18].

In Japan, since the number of AYA patients with cancer at each hospital is small and the primary cancer site varies [19], it is difficult for medical staff to gain experience related to providing medical care and support to AYA patients with cancer. There are marked differences in support for AYA patients with cancer among hospitals [20].

In order to help medical staff identify who is in need of psychosocial intervention and when to intervene most effectively, real-world data about mental health care use and related factors for AYA patients with cancer are needed. However, these data are not well understood in Japan. This study aimed to (1) examine the actual state of mental health care use of AYA patients with cancer and (2) describe socio-demographic and related factors associated with mental health care use.

## Methods

### Participants and procedures

We retrospectively reviewed the medical records of AYA patients with cancer who first visited the National Cancer Center Hospital in Japan (NCCH) between January 2018 and December 2020. NCCH has a department of psycho-oncology that consists of psychiatrists, psychotherapists, and nurses specializing in liaison consultation. The mental health professionals of this department provide mental health care to NCCH patients. We defined mental health care in this study as any medical care provided to both outpatients and inpatients by the mental health professionals with or without a psychiatric diagnosis. An electronic database was used to identify mental health care use and related factors for AYA patients with cancer.

To more directly examine the factors associated with mental health care use and with cancer incidence, the eligibility criteria were as follows: (1) diagnosis of cancer and between 15 and 39 years at the time of the study; (2) duration from diagnosis of cancer to first NCCH visit of less than 1 year; (3) no history of mental health care use, including at other hospitals; and (4) no use of any mental health care at other hospitals during the study period. The eligibility criteria (3) and (4) were set because we were concerned that if those patients were included in this study, it would include patients who used mental health care for factors other than cancer. In addition, this study was a retrospective medical record survey, which made it difficult to collect information on history of mental health care use including at other hospitals and use of any mental health care at other hospitals during the study period.

The database included demographic variables such as age, gender, social status, cancer type (primary site), cancer stage, days from diagnosis of cancer to first NCCH visit, treatment setting (at 1 month after the first visit), treatment type (surgery, radiation, chemotherapy, or hematopoietic stem cell transplantation), smoking, drinking, having spouses or partners, having children, living status, and mental health care use less than 1 year after diagnosis of cancer.

For patients who used mental health care less than 1 year after diagnosis of cancer, the database also included demographic variables such as psychiatric diagnoses according to the Diagnostic and Statistical Manual of Mental Disorders, 5th edition (DSM-V) [21], days from each diagnosis of cancer to mental health care use, treatment setting (where they used mental health care), occupation of mental health care providers, type of medication (sleeping pills, anxiolytic, antipsychotic, antidepressant, or other), number of mental health care visits, and outcome. We defined Group A as patients aged 15–24 years (adolescents and older adolescents) and Group YA as patients aged 25–39 years (young adults) based on the definition of a previous study in Japan [19]. Regarding the content of mental health care use, Groups A and YA were analyzed separately to clarify the differences among them.

This study was approved by the NCCH Ethics Committee (approval number: 2019-215). The requirement for informed consent was waived due to the retrospective cohort design. Opt-out information was published on the NCCH website. This study was conducted in accordance with the principles of the Helsinki Declaration.

### Sample size calculations

We planned to perform a multiple regression analysis to examine the factors related to mental health care use. We calculated that more than 10 times as many study participants as the number of independent variables would be required [22]. Considering the possibility of missing data, the planned number of study participants was more than 200.

### Statistical analysis

First, we used descriptive statistics. Second, the prevalence of mental health care use among all participants was calculated. Third, odds ratios (ORs) and 95% confidence intervals (CIs) were calculated with logistic regression to examine associations between demographic variables and the prevalence of mental health care use. All demographic variables were entered as independent variables. The association between patients' social background characteristics and mental health care use was analyzed to help identify which patients might benefit from early mental health intervention. The association between the patient's course of cancer treatment and mental health care use was analyzed. A forced entry method was used to explore factors related to mental health use. In the logistic regression analysis, cases with even one missing value were excluded from the analysis. Fourth, to compare the socio-demographic and clinical characteristics of patients in Groups A and YA, the chi-square test or Fisher's exact test was used, as appropriate. Data were analyzed with SPSS version 27.0 (IBM). All tests were two-tailed, with a *p*-value of <0.05.

## Results

### Patient characteristics

**Fig. 1:**
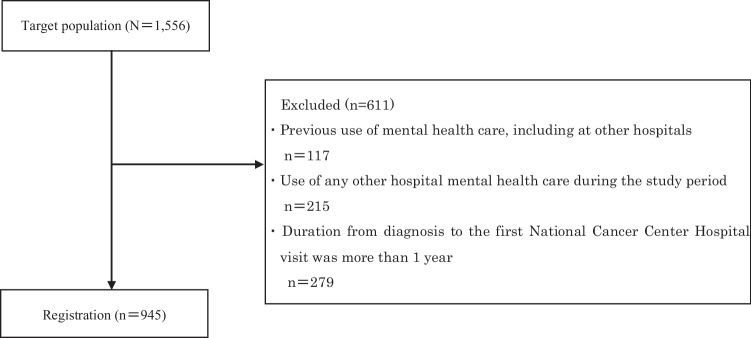
Study flow diagram

Among 1,556 patients, 945 AYA patients with cancer were registered in the database in August 2021 (Fig. 1). The socio-demographic and clinical characteristics of the patients are shown in Table 1. Median age at the time of the study was 33 years (range, 15–39 years). Cancer diagnoses included gastrointestinal cancer (*n*=171; 18.1%), breast cancer (*n*=162; 17.1%), lung cancer (*n*=124; 13.1%), gynecological cancer (*n*=86; 9.1%), head and neck cancer (*n* =84; 8.9%), and other (*n* =318; 33.7%). The most common stage at diagnosis was stage I (*n*=207, 21.9%), followed by stage II (*n*=129, 13.6%). The most common treatment type was curative (*n*=438, 46.4%). The prevalence of mental health care use was 18.0% (170/945).

**Table 1:** Socio-demographic and clinical characteristics of the study participants

	All		Mental health care use			
	(*n*=945)		Yes (*n*=170)		No (*n*=775)	
	N	%	N	%	N	%
Age, years						
15–19	64	6.8	41	64.1	23	35.9
20–24	91	9.6	20	22.0	71	78.0
25–29	136	14.4	33	24.3	103	75.7
30–34	247	26.1	35	14.2	212	85.8
35–39	407	43.1	41	10.1	366	89.9
Gender						
Female	550	58.2	83	15.1	467	84.9
Male	395	41.8	87	22.0	308	78.0
Has spouses or partners						
Yes	523	55.3	65	12.4	458	87.6
No	147	15.6	67	45.6	80	54.4
Unknown	275	29.1	38	13.8	237	86.2
Has children						
Yes	292	30.9	37	12.7	255	87.3
No	649	68.7	132	20.3	517	79.7
Unknown	4	0.4	1	25.0	3	75.0
Living status						
Living together	746	78.9	146	19.6	600	80.4
Living alone	199	21.1	24	12.1	175	87.9
Smoking						
Yes	104	11.0	17	16.3	87	83.7
No	839	88.8	153	18.2	686	81.8
Unknown	2	0.2	0	0.0	2	100.0
Drinking						
Yes	508	53.8	62	12.2	446	87.8
No	434	45.9	108	24.9	326	75.1
Unknown	3	0.3	0	0.0	3	100.0
Employment status						
Employed or student	727	76.9	140	19.3	587	80.7
Unemployed	180	19.1	29	16.1	151	83.9
Unknown	38	4.0	1	2.6	37	97.4

Cancer type (primary site)						
Digestive system	171	18.1	25	14.6	146	85.4
Breast	162	17.1	6	3.7	156	96.3
Lung	124	13.1	29	23.4	95	76.6
Gynecological system	86	9.1	14	16.3	72	83.7
Head and neck	84	8.9	14	19.0	68	81.0
Bone or soft tissue	76	8.0	27	35.5	49	64.5
Urogenital system	65	6.9	12	18.5	53	81.5
Hematological	61	6.5	23	37.7	38	62.3
Skin	45	4.8	3	6.7	42	93.3
Brain	40	4.2	5	12.5	35	87.5
Other	31	3.3	10	32.3	21	67.7
Stage						
0	71	7.5	3	4.2	68	95.8
I	207	21.9	19	9.2	188	90.8
II	129	13.6	16	12.4	113	87.6
III	94	10.0	14	14.9	80	85.1
IV	116	12.3	31	26.7	85	73.3
Unknown (including hematological cancer)	328	34.7	87	26.5	241	73.5
Days from diagnosis to first visit						
Before diagnosis	201	21.3	42	20.9	159	79.1
~3 months	646	68.3	104	16.1	542	83.9
3 months–1 year	98	10.4	24	24.5	74	75.5
Treatment type at 1 month after the first medical visit						
Curative	438	46.4	88	20.1	350	79.9
Palliative	187	19.8	62	33.2	125	66.8
Best supportive care	2	0.2	1	50.0	1	50.0
Other	318	33.7	19	6.0	299	94.0
Treatment						
Surgery						
Yes	420	44.4	49	11.7	371	88.3
No	525	55.6	121	23.0	404	77.0
Radiation						
Yes	191	20.2	38	19.9	153	80.1
No	754	79.8	132	17.5	622	82.5
Chemotherapy						
Yes	454	48.0	126	27.8	328	72.2
No	491	52.0	44	9.0	447	91.0
Hematopoietic stem cell transplantation						
Yes	28	3.0	23	82.1	5	17.9
No	917	97.0	147	16.0	770	84.0

### Factors associated with mental health care use

We used logistic regression to compare socio-demographic and medical factors between patients with and without mental health care use. The association between patients' social background characteristics and mental health care use was analyzed in 426 patients with no missing values. Age 15–19 years, female gender, urogenital cancer, gynecological cancer, bone or soft tissue cancer, head and neck cancer, and stage II–IV disease were associated with mental health care use (Table 2). The association between the patient's course of cancer treatment and mental health care use was analyzed in 627 patients with no missing values. Regarding treatment type, palliative treatment, chemotherapy, and hematopoietic stem cell transplantation were associated with mental health care use (Table 3).

**Table 2:** Patient characteristics associated with mental health care use

	Odds ratio (95% confidence interval)
Age, years	
15–19	11.2 (2.23–56.8) ^**^
20–24	0.87 (0.16–4.61)
25–29	2.43 (0.93–6.32)
30–34	0.99 (0.44–2.23)
35–39	1.00 (Reference)
Gender	
Female	3.20 (1.34–7.68) ^**^
Male	1.00(Reference)
Has spouses or partners	
Yes	0.48 (0.18–1.29)
No	1.00 (Reference)
Has children	
Yes	0.75 (0.33–1.69)
No	1.00 (Reference)
Living status	
Living together	1.17 (0.37–3.71)
Living alone	1.00 (Reference)
Smoking	
Yes	1.46 (0.47–4.50)
No	1.00 (Reference)
Drinking	
Yes	0.90 (0.44–1.86)
No	1.00(Reference)
Employment	
Employed or student	1.33 (0.56–3.18)
Unemployed	1.00 (Reference)
Cancer type (primary site)	
Digestive system	2.78 (0.73–10.6)
Breast	1.00(Reference)
Lung	3.89 (0.94-16.1)
Gynecological system	6.19 (1.61-23.8) ^**^
Head and neck	4.64 (1.03-20.9) ^*^
Bone or soft tissue	14.0 (2.90-67.3) ^**^
Urogenital system	8.15 (1.50-44.2) ^*^
Hematological	1.56 (0.22–11.0)
Skin	2.84 (0.39-20.5)
Brain	19.7 (0.13-2985)
Other	0.00(0.00)

Stage	
0	0.00 (0.00)
I	1.00 (Reference)
II	3.24 (1.15–9.14) ^*^
III	3.05 (1.11–8.37) ^*^
IV	3.88 (1.48–10.2) ^**^
Days from diagnosis to first visit	
Before diagnosis	1.69 (0.79–3.60)
~3 months	1.00 (Reference)
3 months–1 year	1.20 (0.11–13.1)

*p < 0.05

**p < 0.01

***p < 0.001

**Table 3:** Cancer treatment-related factors associated with mental health care use

	Odds ratio (95% confidence interval)
Treatment setting at 1 month after the first medical visit	
Curative	1.00 (Reference)
Palliative	2.03 (1.31–3.15) ^**^
Best supportive care	7.24 (0.43–12.1)
Treatment	
Surgery	
Yes	0.85 (0.54–1.34)
No	1.00(Reference)
Radiation	
Yes	0.65 (0.40–1.07)
No	1.00 (Reference)
Chemotherapy	
Yes	2.38 (1.52–3.74) ^***^
No	1.00 (Reference)
Hematopoietic stem cell transplantation	
Yes	18.4 (6.50–52.2) ^***^
No	1.00 (Reference)

*p < 0.05

**p < 0.01

***p < 0.001

### Mental health care use in AYA patients with cancer

Details about mental health care use are shown in Table 4. Among 170 patients who used mental health care, 71.8% (*n*=122) of patients did not have a psychiatric diagnosis. The most common duration from diagnosis of cancer to mental health care use was less than 3 months (*n*=103, 60.6%). The most common treatment type was curative (*n*=92, 54.1%). The most common occupation who provided support was psychotherapist (*n*=132, 77.6%). Among 75 patients who were prescribed psychotropic drugs, the most common psychotropic drug type was sleeping pills (*n*=34, 45.3%). The most common number of visits ranged from 2 to 5 (*n*=62, 36.5%). The most common outcome was improvement (*n*=132, 77.6%).

**Table 4:** Details about mental health care use

	All		Adolescents and older adolescents (Age 15–24 years)		Young adults (Age 25–39 years)		χ2	*p*
	*N*	%	*N*	%	*N*	%		
Psychiatric diagnosis							15.9	<0.001
No	122	71.8	55	90.2	67	61.5		
Yes	48	28.3	6	9.8	42	38.5		
Sleep-wake disorders	19	11.2	1	16.7	18	42.8		
Trauma-related and stressor-related disorders	14	8.2	2	33.3	12	28.5		
Neurocognitive disorders	6	3.5	0	0.0	6	14.3		
Neurodevelopmental disorders	4	2.4	2	33.3	2	4.8		
Depressive disorders	2	1.2	0	0.0	2	4.8		
Anxiety disorders	2	1.2	1	16.7	1	2.4		
Somatic symptom and related disorders	1	0.6	0	0.0	1	2.4		
Duration from diagnosis to mental health care use								0.116 ^a^
Before diagnosis	3	0.02	1	0.02	2	0.02		
~3 months	103	0.60	43	0.70	60	0.55		
3 months–1 year	64	0.38	17	0.28	47	0.43		
Treatment setting at the time of mental health care use								0.086 ^a^
Curative treatment	92	54.1	39	63.9	53	48.6		
Palliative treatment	70	41.2	22	36.1	48	44.0		
Before treatment	5	2.9	0	0.0	5	4.6		
Observation	3	1.8	0	0.0	3	2.8		
Occupation who supported (with duplicates)							9.7	0.008
Psychotherapist	132	77.6	56	91.8	76	69.7		
Psychiatrist	126	74.1	34	55.7	92	84.4		
Nurse specializing in liaison consultation	34	20.0	7	11.5	27	24.8		
Medication type (with duplicates)								0.810 ^a^
Sleeping pills	34	45.3	2	3.3	32	29.4		
Anxiolytics	19	25.3	1	1.6	18	16.5		
Antipsychotics	11	14.7	0	0.0	11	10.1		
Antidepressants	9	12.0	1	1.6	8	7.3		
Other	2	2.7	0	0.0	2	1.8		
Number of times that mental health care were used							0.76	0.858
1	36	21.2	13	21.3	23	21.1		
2–5	62	36.5	23	37.7	39	35.8		
6–9	27	15.9	11	18.0	16	14.7		
≥10	45	26.4	14	23.0	31	28.4		
Outcome								0.106 ^a^
Improvement	132	77.6	51	83.6	81	74.3		
Death	16	9.4	7	11.5	9	8.3		
Under treatment	9	5.3	3	4.9	6	5.5		
Hospital transfer	9	5.3	0	0.0	9	8.3		
Self-interruption	3	1.8	0	0.0	3	2.7		
Other (under treatment at another hospital)	1	0.6	0	0.0	1	0.9		

^a^Fisher's exact test

We compared the socio-demographic and clinical characteristics of the patients in Groups A and YA using the chi-square test and Fisher's exact test as appropriate. Group A had a significantly higher percentage of patients with no psychiatric diagnosis relative to Group YA. A significantly higher percentage of patients in Group A was supported by psychotherapist, whereas a significantly higher percentage of patients in Group YA was supported by psychiatrist. Consistent with this, Group YA had more patients who were treated with medication than Group A.

## Discussion

In this study, we examined the actual state of mental health care use of AYA patients with cancer and described the socio-demographic and related factors associated with mental health care use in Japan. This study has several strengths. First, our findings suggested that urogenital cancer, gynecological cancer, bone or soft tissue cancer, and palliative treatment were associated with mental health care use, which had not been previously reported. Second, there were more patients with sleep-wake disorders in this study than in a previous study [37], followed by trauma-related and stressor-related disorders. These new points should be noted when supporting AYA patients with cancer. Finally, our findings suggested that age 15–19 years, female gender, head and neck cancer, stage II–IV disease, chemotherapy, and hematopoietic stem cell transplantation are associated with mental health care use. Although these findings were consistent with those of previous studies, it was important to note that these data were from the real world, not questionnaire surveys.

The prevalence of mental health care use in AYA patients with cancer (age range, 15–39 years) in this study was 18.0% (170/945). On the other hand, although the data were from survivors, not patients, approximately 30% of cancer survivors in Japan (age range: 34–79 years) reportedly use mental health care [23]. Although our findings suggest that mental health care use among AYA patients with cancer is lower than among other generations, this does not mean that AYA patients with cancer have a lower need for mental health care. In Japan, more than 70% of AYA patients with cancer reported unmet supportive care needs, mostly psychological [24]. These findings suggest that AYA patients with cancer have unmet mental health care needs. This is consistent with the findings of a previous study [6]. We need to work on addressing the current gaps in mental health care delivery.

Age 15–19 years and female gender were patient characteristics associated with mental health care use. Although correlations between age and psychological distress in previous studies were inconsistent [10, 13], younger age (15–19 years) was associated with mental health care use in this study. This finding was consistent with that of a previous study in China [13]. In younger AYA patients with cancer, the body and mind are still growing and developing; younger AYA patients with cancer might have special needs and challenges in fertility preservation, parenting, schooling, and employment compared to patients of other ages. Therefore, we need to pay much more attention to younger AYA patients with cancer. Parents have described their adolescent's cancer-related distress as the most problematic symptom, contributing to symptom burden on both adolescent patients and their parents [25]. Therefore, parental concerns might have led to mental health care use by adolescents with cancer. Female gender was associated with mental health care use. This was consistent with the finding that female gender is associated with psychological distress [12]. Female patients with cancer were reported to be more affected by depression and anxiety [26]; this tendency might lead to mental health care use more than in males.

Urogenital cancer, gynecological cancer and bone or soft tissue cancer were associated with mental health care use in this study, but were not associated with mental health care use in from a previous study [13]. This difference might be related to the impact of cancer incidence and cancer treatment on fertility. In gynecologic and urologic cancers, the cancer has a direct effect on fertility. In bone or soft tissue cancer, the risk of chemotherapy affecting fertility has been reported [27, 28]. Patients with cancer in Japan request information about fertility preservation and psychological support [29]. However, a system for providing explanatory materials for fertility preservation and encouraging cooperation at the physician and hospital levels are insufficient support for AYA patients with cancer [30]. Therefore, these cancer types might be related to mental health care use. Head and neck cancer was a related factor, as in a previous study [13]. The prevalence of depression in patients with head and neck cancer is higher than in other types of cancer [31, 32]. Head and neck cancer is a strong risk factor for suicide [33]. Patients with head and neck cancer are prone to significant psychological strain due to pain, dysfunction, compression by the tumor, communication difficulties, and changes in appearance, in addition to the general psychological problems associated with cancer. It was considered a type of cancer that requires adequate attention in terms of psychological care in the AYA generation.

Stage II–IV disease was associated with mental health care use. As the stage increased, so did the OR for mental health use. In general, the higher the stage, the worse the prognosis for cancer, so this result is reasonable. These findings were consistent with the presence of a significant association between depression and advanced or metastatic cancer [34].

Palliative treatment was associated with mental health care use. Although not reported in previous studies, it is easy to imagine patients feeling depressed because there is no radical cure to be sought.

Chemotherapy and hematopoietic stem cell transplantation were associated with mental health care use. These were consistent with the findings of previous studies [13, 35]. It has been reported that patients with cancer experience anxiety and depression during chemotherapy [36]. Many AYA patients with cancer who undergo hematopoietic stem cell transplantation have described unmet psychological needs [35].

Details about mental health care use were compared with the results of a previous study of patients with cancer in Japan [37]. In this study, there were more patients with sleep-wake disorders (39.6%, 19/48) than in that previous study, but fewer patients with delirium, which is included in the category of neurocognitive disorders. Various studies [38–41] have shown that delirium in patients with cancer is associated with older age, which is consistent with our findings with younger patients. Consistent with the high prevalence of sleep-wake disorders, sleeping pills were the most frequently prescribed medication. Because of the high rate of sleep disturbances, we need to pay close attention to the sleep of AYA patients with cancer.

After sleep-wake disorders, the most common diagnosis was trauma-related and stressor-related disorder (29.2%, 14/48). The most common trauma-related and stressor-related disorder diagnosis was adjustment disorder. A meta-analysis reported that psychotherapy is effective in patients with cancer with depressive symptoms, including those with adjustment disorders [34]. Psychotherapy is a specialty of the psychotherapist, the most common occupation who provided support in this study. This result suggests that the psychotherapist is essential in the mental health care of AYA patients with cancer. In Japan, even large hospitals have shortages of psycho-oncologists [19]. Therefore, psychotherapists might play a role in filling these shortages.

In Group YA, 61.5% of patients had no psychiatric diagnoses whereas 90.2% of patients in Group A had no psychiatric diagnoses. In Group YA, 69.7% of patients were supported by a psychotherapist, compared with 91.8% of patients in Group A. These results suggest that there is a great need for psychotherapists to treat adolescents. This may be due to the fact that psychotherapists provided support for psychological reactions after bad news at NCCH.

This study has several limitations. First, this study had a single-center retrospective cohort design. Results might not be generalizable to other settings because the NCCH support system for AYA patients with cancer was robust [42–45]. Further prospective multicenter studies are needed. Second, all data were collected retrospectively from medical records. The possibility of out-of-hospital mental health care use not documented by the health care provider cannot be ruled out. Third, there were several items that the information in the medical records did not reveal. Because cases with even one missing value were excluded from the analysis in the logistic regression analysis, the results in Tables 1, 2, and 3 were partially inconsistent. For example, although male had a higher percentage of mental health use in Table 1, it was female who were associated with mental health use in the logistic regression analysis. Furthermore, the final number of patients used in the logistic analysis was much smaller than the overall number of patients. The exclusion of cases with any missing value from the analysis may have biased the results. Fourth, because this study was a retrospective medical record survey, with many missing values and a small number of subjects for analysis in logistic regression, this study was unable to examine any difference in the related factors associated with mental health care use between Groups A and YA. Finally, low income was known to be associated with psychological distress in AYA cancer patients [10, 13, 14]. However, items related to economic aspects were not extracted in this study, as it was assumed that such items would be experientially less likely to be included in the medical records of NCCH. The next challenge is to focus on the economic aspects of AYA cancer patients. Finally, study participants were all Japanese. Careful consideration might be required when generalizing our findings to other races and ethnic groups. Despite these limitations, our findings potentially contribute to interventions that provide psychological support to AYA patients with cancer.

## Conclusions

Factors associated with mental health care use were identified. Our findings potentially contribute to interventions that provide psychological support to AYA patients with cancer.

## Data Availability

The data that support the findings of this study are available from the corresponding author upon reasonable request.
